# Supplementary material for: Integrated Proteomics and Metabolomics Link Acne to the Action Mechanisms of Cryptotanshinone Intervention
Source: Front Pharmacol. 2021 Sep 1;12:700696. doi: 10.3389/fphar.2021.700696 (PMC8440807; doi:10.3389/fphar.2021.700696)
Supplement: Supplementary file 1 [file DataSheet1.ZIP › Supplementary table 1-10 and material 1-3/Material 3-Metlib-MSMS/POS-Metlib-MSMS/Metlib-MSMS/M113T163_2_forward/0.966,Uracil,(M+H)+.pdf]

Uracil

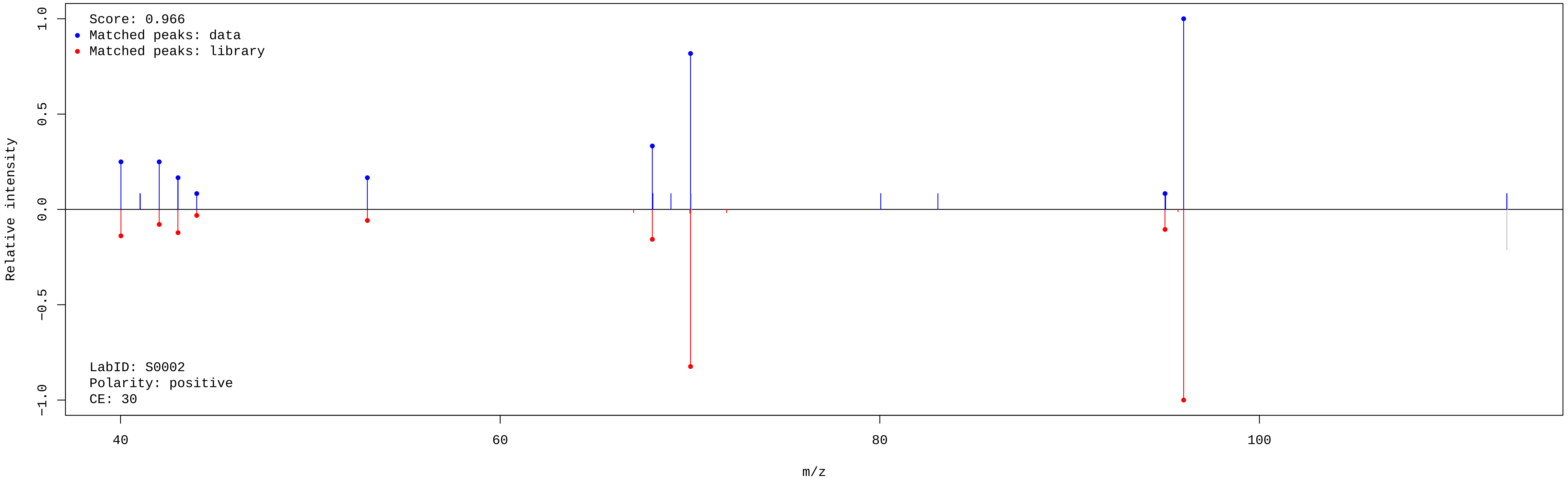

Score: 0.966
Matched peaks: data
Matched peaks: library
LabID: S0002
Polarity: positive
CE: 30
Relative intensity
m/z
1.0
0.5
0.0
−0.5
−1.0
40
60
80
100
